# Supplementary material for: Discrete choice experiment versus swing-weighting: A head-to-head comparison of diabetic patient preferences for glucose-monitoring devices
Source: PLoS One. 2023 Jul 28;18(7):e0283926. doi: 10.1371/journal.pone.0283926 (PMC10381030; doi:10.1371/journal.pone.0283926)

**Appendix I: Example choice task**

Imagine that your doctor told you to check your blood glucose levels at least four times per day. To do this, the doctor offers you different hypothetical devices to choose from.

|  | **Device A** |  | **Device B** |
| --- | --- | --- | --- |
| **Precision compared to fingerpricking** | Less accurate than fingerpricking (higher or lower by 0.3) |  | Less accurate than fingerpricking (higher or lower by 0.6) |
| **Average number of fingerpricks per day** | 0 |  | 0 |
| **Effort to check** | Low effort |  | Moderate effort |
| **Probability of getting skin irritation or redness** | 5% chance of skin irritation or redness (5 out of 100) |  | 35% chance of skin irritation or redness (35 out of 100) |
| **Glucose information** | Current Glucose level |  | Current Glucose level and arrow |
| **Alarms** | Yes |  | No |
| **Monthly costs** | €25 |  | €175 |
| **I prefer:** | 🔾 |  | 🔾 |

If you have to choose between the device you have chosen above and the traditional fingerprick-test to check your glucose levels, which one would you prefer? (Please note that a fingerprick-test should be done four times a day, requires high effort to check, does not result in skin irritation or redness, will show your glucose levels, doesn’t have an alarm and costs €25 per month).

*Select only one answer*

| 🔾 **I prefer the device I have selected above** | 🔾 **I prefer the fingerprick-test** |
| --- | --- |

**Appendix II: Swing weighting**

**Part 1: Ranking**

If you could improve one characteristic of a glucose monitor from being the worst possibility to the best possibility, which would you improve?

Click on the characteristic that you would like to improve first.

Click on the characteristic that you would want to improve next. Continue until all the characteristics have disappeared.

Note: You can hover your mouse over each characteristic to learn more about it

**Which would you choose first?**

| **Glucose information:**  ***Current glucose level ➔  Current glucose level and a graphic of your level trends over the day*** | 🔾 |
| --- | --- |
| **Effort to check: *High effort (you need to measure your glucose levels yourself) ➔ Low effort (glucose levels automatically sent to you)*** | 🔾 |
| **Probability of getting skin irritation or redness: *35% chance (35 out of 100) ➔ No chance*** | 🔾 |
| **Alarms:**  ***No ➔  Yes*** | 🔾 |
| **Average number of fingerpricks per day: *4 daily  ➔  0 daily*** | 🔾 |
| **Out of pocket cost per month: *€250➔  €25*** | 🔾 |
| **Precision compared to fingerpricking: *Less accurate than fingerpricking (higher or lower by 0.6) ➔  Accurate as fingerpricking*** | 🔾 |

| Restart selection |
| --- |

**Part 2: Point allocation**

Imagine that improving [participant’s first choice] is worth 100 points. Please give points to the other characteristics based on how important you think improving them would be compared to improving [participant’s first choice].

You can give any number of points to each improvement from 0 (not at all important) to 100 (just as important as [participant’s first choice]). For example, if you give 50 points to an improvement, it means that you think it is half as important as [participant’s first choice] because you gave it half as many points.

| **[Participant’s 1st choice]** | 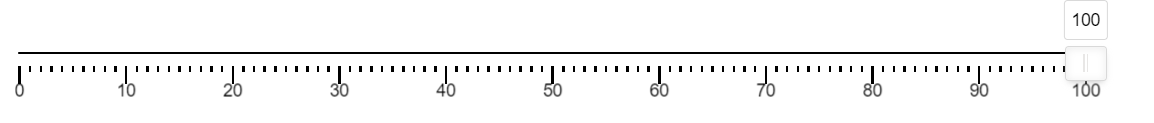 |
| --- | --- |
| **[Participant’s 2nd choice]** | 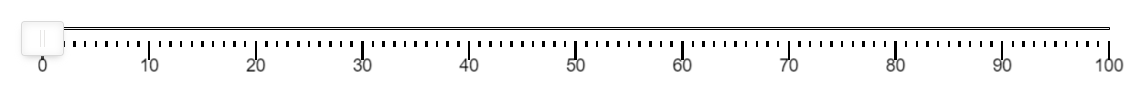 |
| **[Participant’s 3rd choice]** | 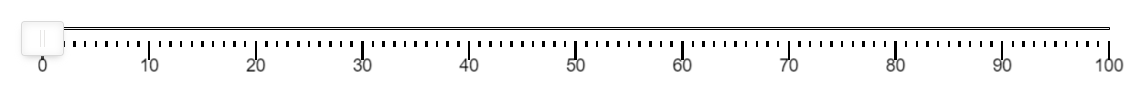 |
| **[Participant’s 4th choice]** | 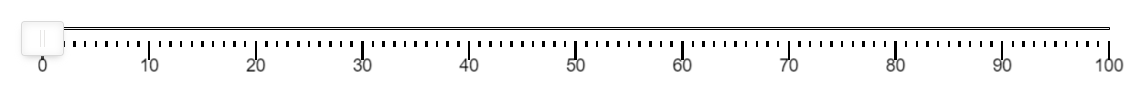 |
| **[Participant’s 5th choice]** | 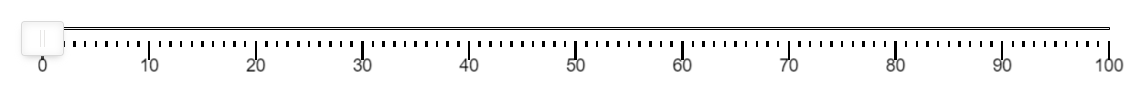 |
| **[Participant’s 6th choice]** | 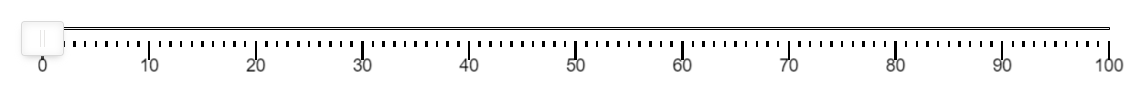 |
| **[Participant’s 7th choice]** | 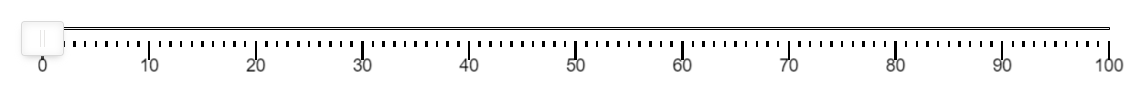 |

**Appendix III: Ethical Approval**

On 17 December 2019, this study (Reference number WAG/mb/19/045208) was granted approval by the Medical Research Ethics Committee, UMC Utrecht and confirmed that the Medical Research Involving Human Subjects Act 1998 (Wet Medisch-Wetenschappelijk Onderzoek Met Mensen (WMO)) does not apply to the study because (1) it does not concern medical scientific research and (2) participants are not subject to procedure or are required to follow rules of behaviour. The study was conducted according to the principles of the Declaration of Helsinki.

**Appendix IV: Respondent characteristics (n=459)**

| **Characteristics** | **All patients n=459†** |
| --- | --- |
| Age in years  (mean ± sd) | 51.0 ± 17.5 |
| Sex (n, %)  Females  Males | 233 (50.8)  225 (49.0) |
| Glucose monitor currently used  CGM or FGM  Fingerprick-testing only  None | 84 (18.3)  250 (54.4)  125 (27.2) |
| Type of diabetes (n, %)  Type 1  Type 2  Other | 124 (27.0)  317 (69.1)  18 (3.9) |
| Educational level ^a^ (n, %)  High  Intermediate  Low | 184 (40.1)  244 (53.2)  31 (6.8) |

CGM= continuous glucose monitor; FGM = ‘Flash’ glucose monitor

(a) High represents Bachelors, masters, or higher degree; Intermediate represents general secondary education, vocational secondary education, or gymnasium; Low represents lower secondary (high school), primary school, or no education.

†Of 5,620 invited participants, 500 completed the survey, indicating a response rate of 8.9%.

**Appendix V: Comparison of the probability that an attribute has a certain rank between DCE and SW (Table)**

|  |  | **Attributes** | | | | | | |
| --- | --- | --- | --- | --- | --- | --- | --- | --- |
| **Probability of Attribute Having Certain Ranks 1-7** | **Method** | Precision compared to fingerpricking† | Average number of fingerpricks per day† | Effort to check† | Glucose information‡ | Probability of getting skin irritation or redness‡ | Monthly costs† | Alarms‡ |
| Rank 1 (highest) | DCE | 0.34 | 0.47 | 0.11 | 0.04 | 0.09 | 0.03 | 0.03 |
|  | SW | 0.17 | 0.19 | 0.09 | 0.09 | 0.02 | 0.23 | 0.08 |
| Rank 2 | DCE | 0.02 | 0.22 | 0.43 | 0.04 | 0.24 | 0.12 | 0.03 |
|  | SW | 0.20 | 0.22 | 0.13 | 0.08 | 0.07 | 0.13 | 0.06 |
| Rank 3 | DCE | 0.01 | 0.10 | 0.17 | 0.09 | 0.34 | 0.19 | 0.04 |
|  | SW | 0.15 | 0.17 | 0.14 | 0.14 | 0.20 | 0.16 | 0.09 |
| Rank 4 | DCE | 0.01 | 0.06 | 0.13 | 0.15 | 0.16 | 0.39 | 0.05 |
|  | SW | 0.17 | 0.15 | 0.14 | 0.20 | 0.21 | 0.12 | 0.11 |
| Rank 5 | DCE | 0.002 | 0.08 | 0.07 | 0.38 | 0.09 | 0.12 | 0.10 |
|  | SW | 0.11 | 0.10 | 0.18 | 0.33 | 0.21 | 0.11 | 0.17 |
| Rank 6 | DCE | 0.002 | 0.05 | 0.06 | 0.21 | 0.04 | 0.09 | 0.39 |
|  | SW | 0.12 | 0.11 | 0.18 | 0.12 | 0.15 | 0.10 | 0.33 |
| Rank 7 (lowest) | DCE | 0.62 | 0.02 | 0.03 | 0.09 | 0.03 | 0.06 | 0.37 |
|  | SW | 0.08 | 0.06 | 0.14 | 0.04 | 0.13 | 0.17 | 0.14 |
| Number of observations |  | 918 | 918 | 918 | 918 | 918 | 918 | 918 |
| Wald chi2 |  | 444.98 | 89.88 | 156.02 | 31.78 | 127.53 | 156.97 | 84.40 |
| p-value |  | <0.0001 | <0.0001 | <0.0001 | <0.0001 | <0.0001 | <0.0014 | <0.0001 |

DCE = Discrete choice experiment; SW = Swing weighting.

†Based on generalized ordered logit analyses; ‡Based on ordered logit analyses because the generalised ordered logit did not converge

**Appendix VI: Comparison of the probability that an attribute has a certain rank between DCE and SW (Figure)**


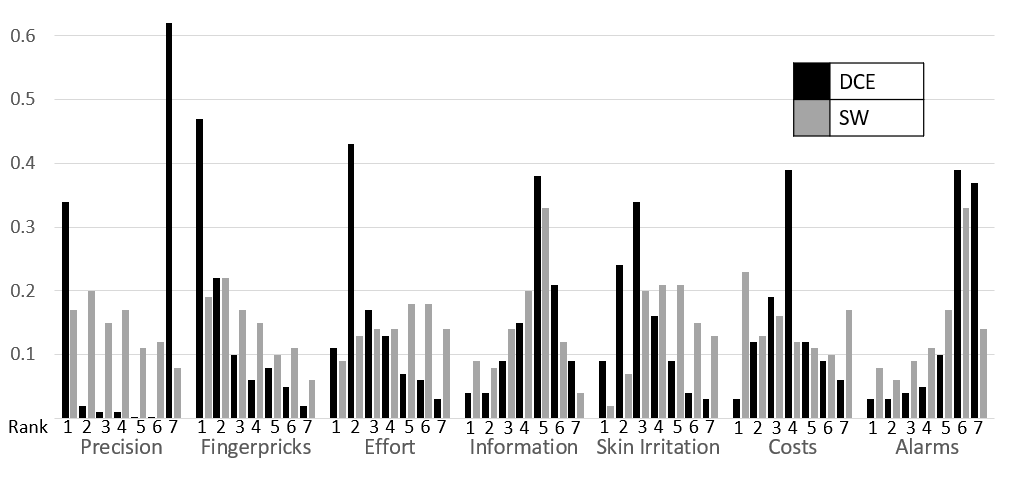

Supplement: S1 File — (DOCX) [file pone.0283926.s001.docx]
