# Supplementary material for: Discrete choice experiment versus swing-weighting: A head-to-head comparison of diabetic patient preferences for glucose-monitoring devices
Source: PLoS One. 2023 Jul 28;18(7):e0283926. doi: 10.1371/journal.pone.0283926 (PMC10381030; doi:10.1371/journal.pone.0283926)
Supplement: Attachment — Submitted filename: PONE-D-21-37787 Response to Reviewers 17Mar2023.docx [file pone.0283926.s003.docx]

Dear PlosOne Editors,

We would like to re-submit our PONE-D-21-37787 paper entitled ‘Discrete choice experiment versus swing-weighting: A head-to-head comparison of diabetic patient preferences for glucose-monitoring devices’ to PlosOne, because we believe this article could be of great interest for you and your readers. We sincerely appreciate the minor revision requested from your reviewer, and we would like to integrate and address it in this resubmission.

1) Reviewer #1: Thanks the authors to provide revisions to the manuscript which addresses most of the comments. There is one more comment from me. For sensitivity analysis for attribute rankings between DCE and SW, while the authors have added a few details to its method, it is still unclear how respondent-level ranking of attributes was derived from DCE. Could the authors tell more specifically how to use DCE marginal utility (which I assumed refers to model estimates in Table 2) to find out the attribute rankings of each individual respondent?
   i. Response: Thank you for this comments, and we have provided more detail the heading “sensitivity analyses” for the methodology of (generalized) ordered logit model. We hope that this explanation provides greater clarification:

*“The respondent-level ranking of the attributes in the DCE and the SW were identified by determining how each individual participant ranked the attributes, either by participant rankings for the SW, or a ranked marginal utility for the DCE method. These individual ranking were then compared using a (generalised) ordered logit model. For the SW, these were individual-specific ranking outputs derived from Step 1 of the SW method, ranking the attributes from 1 to 7. For the DCE, the individual-specific rankings of each attribute were determined by first examining the patient uptake rates for the most preferred device (high precision, zero fingerpricks, low effort, low skin irritability, 25 euro, plain information, no alarm) replicating the same device hypothetically created during the SW exercise (i.e. the swing from ‘worst’ to ‘best’ attributes). As detailed above, the systematic utility is defined as an additive function consisting of marginal utilities. Therefore, the coefficients for each corresponding attribute-level for each individual were then ranked from lowest to highest and given a corresponding value (1 to 7). Each participant’s DCE rankings were then compared against their SW rankings through the ordered logit model, and determining* *the probability that an attribute had of being ranked 1-7 in either the DCE or the SW.”*

We sincerely hope that this submission will be accepted upon this resubmission. Thank you for your attention, and looking forward to hearing from you,

Chiara Lauren Whichello, PhD
